# Benefits of Instructed Responding in Manual Assembly Tasks: An ERP Approach

**DOI:** 10.3389/fnhum.2016.00171

**Published:** 2016-04-20

**Authors:** Pavle Mijović, Vanja Ković, Maarten De Vos, Ivan Mačužić, Branislav Jeremić, Ivan Gligorijević

**Affiliations:** ^1^Department for Production Engineering, Faculty of Engineering, University of KragujevacKragujevac, Serbia; ^2^Laboratory for Neurocognition and Applied Cognition, Department for Psychology, Faculty of Philosophy, University of BelgradeBelgrade, Serbia; ^3^Department of Engineering, The Institute of Biomedical Engineering, University of OxfordOxford, UK

**Keywords:** neuroergonomics, wireless electroencepholagraphy, event-related potentials, P300, attention, manual assembly

## Abstract

The majority of neuroergonomics studies are focused mainly on investigating the interaction between operators and automated systems. Far less attention has been dedicated to the investigation of brain processes in more traditional workplaces, such as manual assembly, which are still ubiquitous in industry. The present study investigates whether assembly workers’ attention can be enhanced if they are instructed with which hand to initiate the assembly operation, as opposed to the case when they can commence the operation with whichever hand they prefer. For this aim, we replicated a specific workplace, where 17 participants in the study simulated a manual assembly operation of the rubber hoses that are used in vehicle hydraulic brake systems, while wearing wireless electroencephalography (EEG). The specific EEG feature of interest for this study was the P300 components’ amplitude of the event-related potential (ERP), as it has previously been shown that it is positively related to human attention. The behavioral attention-related modality of reaction times (RTs) was also recorded. Participants were presented with two distinct tasks during the simulated operation, which were counterbalanced across participants. In the first task, digits were used as indicators for the operation initiation (Numbers task), where participants could freely choose with which hand they would commence the action upon seeing the digit. In the second task, participants were presented with arrows, which served as instructed operation initiators (Arrows task), and they were instructed to start each operation with the hand that corresponded to the arrow direction. The results of this study showed that the P300 amplitude was significantly higher in the instructed condition. Interestingly, the RTs did not differ across any task conditions. This, together with the other findings of this study, suggests that attention levels can be increased using instructed responses without compromising work performance or operators’ well-being, paving the way for future applications in manual assembly task design.

## Introduction

The importance of studying the human brain processes while executing everyday complex tasks in naturalistic environments was pinpointed by Parasuraman (2003), through a new direction in human factors and ergonomics (HF/E) research. This novel direction was tentatively named neuroergonomics (Parasuraman, 2003; Parasuraman and Rizzo, 2006; Parasuraman, 2011). Although Parasuraman and Wilson (2008) modestly stated that neuroergonomics should not be thought of as revolutionary, but rather as another step in HFE research, the growing body of neuroergonomics research refutes this statement. In fact, ever advancing technology has facilitated neuroergonomics research and only 12 years from its inception it has become one of the principal directions in HFE research. Ultimately, understanding brain processes in naturalistic environments can lead to improvement of existing industrial processes design and to creation of safer and more efficient working conditions (Parasuraman, 2003), consequently improving the operators’ overall well-being.

Neuroergonomics has had significant success in evaluating brain activity in its interaction with automated systems, through the studies of mental workload, dual-task performance (Ayaz et al., 2013) and operators’ vigilance (Warm and Parasuraman, 2006; Warm et al., 2009). Additionally, it has gone a step further with the development of state-of-the-art neuroadaptive systems facilitating the mutual interaction between an automated system and operators, in the sense that both human and the system can initiate a change in the level of automation when needed (Scerbo, 2006; Mehta and Parasuraman, 2013). On the one hand, this trend is understandable as industry, over several decades, has tried to reach the “lights-out manufacturing” concept (Tompkins et al., 2010), i.e., completely automated factories which can operate without the direct presence of human operators in the production processes. In that case, human supervisory control of automated systems becomes essential (Sheridan and Parasuraman, 2005), as human operators would be solely responsible for controlling the automated production systems (Warm et al., 2008). On the other hand, although automation is becoming ubiquitous in industry and everyday life (Parasuraman and Wilson, 2008), the “lights-out” concept is still rather futuristic and there is still a need for human manual operations in production processes. This is especially notable in assembly tasks and processes where costs related to process automation are generally not justifiable (Tang et al., 2003).

For these reasons, it is evident that neuroergonomics studies should pay additional attention to more traditional workplaces, through investigation of concurrent physical and cognitive work. This approach has received far less attention in neuroergonomic studies (for review see Mehta and Parasuraman, 2013). For example, in the car manufacturing industries the majority of processes are automated, however human operators play a crucial role in the final car cockpit and interior assembly, i.e., final assembly (Michalos et al., 2010). Typically, manual assembly tasks require a large number of repetitions and are monotonous in nature, thus leading to hypo-vigilance of operators (Spath et al., 2012). In turn, operators have difficulty in sustaining the desired level of attention during the task, and therefore, the risk of work-related injuries, material damage or even accidents is increased (Kletz, 2001). Therefore, employing existing neuroimaging techniques to understand the way the brain processes various stimuli in this class of tasks could be beneficial, as the task design could be optimized in such a way as to obtain and maintain sufficient operator attention, thereby avoiding possibly hazardous situations.

An extensive review of neuroimaging techniques applicable to neuroergonomics research has been recently published by Mehta and Parasuraman (2013). Although functional near infrared spectroscopy (fNIRS) presents a convenient technique for the neuroergonomics research in naturalistic setting due to its light weight and portability (Ayaz et al., 2010, 2012; Mehta and Parasuraman, 2013), it suffers from low temporal resolution and its use in dynamic everyday environments is still somewhat limited (Gramann et al., 2011). On the other hand, Electroencephalography (EEG) and therefrom derived event related potentials (ERPs) belong to the neuroimaging techniques that directly measure brain activity (Gramann et al., 2011; Mehta and Parasuraman, 2013) and both EEG and ERPs possesses high temporal resolution (down to the order of milliseconds) making them suitable for real-time investigation of brain dynamics in complex environments (Gramann et al., 2011). Even though Parasuraman (1990) proposed the introduction of ERPs in ergonomics research, until recently the traditional EEG recording suffered from long wiring between the electrode cap and amplifier unit, which engenders the artifacts that degrades signal quality (Debener et al., 2012). Additionally, EEG recordings usually required shielded, dimly lit and sound attenuated rooms, which was one of the main precondition for its recording, thus limiting its use in naturalistic environments (Gramann et al., 2011). However, these problems were recently overcome by the development of wearable EEG systems, empowering its use in everyday and applied settings (Debener et al., 2012; De Vos et al., 2014; Wascher et al., 2014; Mijović et al., 2016). Consequently, operators’ brain dynamics can nowadays be successfully investigated with wearable EEG in faithfully replicated workplaces, by simulating the work activity (Wascher et al., 2014; Mijović et al., 2016). This can provide insight in how the brain responds to complex industrial tasks and these findings can contribute to more efficient task designs.

The aim of this article is the investigation of assemblers’ mental states, by utilizing ERPs in a realistically replicated workplace. Neuroergonomics implies that overt performance measurements are unreliable (Parasuraman, 2003), since they do not provide the possibility for timely investigation of the underlying covert cognitive processes during everyday tasks. To get better insights into the temporal course of the underlying attention processes engaged in manual assembly operation, we selected two tasks in which we triggered goal-directed actions of workers by presenting them with either digits (in one) or arrows (in the other task) prior to initiating the operation. In this way we wanted to elicit the P300 ERP component (also called P3 or P3b), which is represented by the positive ERP voltage deflection that usually appears between 300 and 500 ms after appearance of the task-relevant stimuli (Polich and Kok, 1995; Verleger et al., 2005). The P300 component is often used to identify the depth of cognitive information processing and its amplitude and latency are considered to be related to the human attention level (Johnson, 1988; Polich, 2007; De Vos et al., 2014).

The P300 complex is the most prominent over the midline scalp sites (Polich, 2007) and it is among the most prominent ERP components (Verleger et al., 2014), making it one of the most studied components of human ERP. However, there is still a lack of consensus regarding what brain functions the P300 component represents (the arguments are briefly summarized in Verleger et al., 2014). One influential view is that the P300 component can be explained through the context updating hypothesis that was proposed by Donchin (1981) and which governs that the P3 reflects the updating of working memory that is related to task-relevant and unexpected events. The context updating theory assumes that the mental process that elicit the P3 component reflects a revision of the model of the environment rather than serving to organize a response to the eliciting stimulus (Verleger et al., 2005). In other words, it is assumed that following an initial sensory processing, an attention-related process evaluates the presentation of the previous event in working memory and if a new stimulus in a train of standard stimuli is detected, the attention-related process updates, which is followed by production of the P300 component (Polich, 2007). However, we have also witnessed arguments against the context updating theory (Verleger et al., 2005, 2014). In fact, Verleger et al. (2005) proposed a new hypothesis in which they argued that the P300 component is related both to stimuli processing and organizing the response. In order to prove this hypothesis, Verleger et al. (2005) compared the P3 amplitude in stimulus- and response-locked ERPs and they found that both P3 amplitudes were comparable. Therefore, it was confirmed that P300 amplitude does not reflect just the simple reaction to stimulus change. Rather, P300 reflects a process that mediates between perceptual analysis and response (Verleger et al., 2005), i.e., it is related to the organization of the response and it depends on the stimulus-response links (Verleger et al., 2014).

Based on these findings, the present study investigated whether and how the neural correlates of goal-directed actions would differ if the operators were requested to initiate the simulated assembly operation spontaneously (upon seeing a digit), as opposed to the condition where participants were instructed with which hand to commence the operation (upon seeing an arrow). In the spontaneous condition (the Numbers task), we adopted the stimuli from the original SART paradigm that is a simple “go/no-go” task, which consists of consecutively presenting digits from “1” to “9” and participants are required to give a speedy response on all stimuli, with the exception of digit “3” (Robertson et al., 1997). The main difference between the original SART and the Numbers paradigm (used in our study) is that the digits in Numbers are randomized. Further, in the original SART paradigm it is requested that participants provide the speedy response with the index finger upon the stimulus presentation. However, this would impede the simulation of the real working operation, since it would require an additional, task-unrelated operation from participants. Instead, in the Numbers paradigm, participants were instructed to initiate the assembly operation as soon as the visual (target) stimulus appeared on the screen, with whichever hand they felt more comfortable (the assembly operation is explained in detail in Section “Simulated Assembly Operation”). For the instructed responding (the Arrows) task, we adopted the stimuli and procedures from Donkers and van Boxtel (2004). The Arrows task is essentially a choice reaction task, where the arrows pointing to the left and right appear on the screen; white arrows represent the target (“go”) condition, while red arrows represent the “no-go” stimulus. The main difference between the Numbers and Arrows tasks was that in the Numbers task participants could freely choose the hand with which they would initiate the assembly operation, while in the Arrows task, participants were instructed to commence each operation with the hand that corresponds to the direction in which the white arrow on the screen was pointing. An important notion is that not only the simple stimulus difference between the tasks was varied (digit vs. arrow), but also the informational value of those stimuli: the Arrows task arguably requires stimulus-response mapping, which in turn requires more cognitive evaluation, consequently inducing higher-level attentional processing than in the simple “go/no-go” task. In both the task specific and spontaneous condition, the visual stimuli (digits and arrows) appeared in the center of a screen that was placed in front of the participants.

We expected attention, when assessed through the P300 amplitude, to be more enhanced in the instructed responding (Arrows) task, compared to the one where participants could initiate the assembly operation upon seeing the task unspecific cue (Numbers task). Further, we wanted to investigate whether the difference in the task condition would also influence the reaction times (RTs), as the performance of the participants is also important, since this study simulates the naturalistic assembly task replicated from the industry. In other words, we wanted to investigate whether the participants would be slower in the case when they are instructed with which hand they should start the assembly operation, as compared to the condition when they can spontaneously initiate the assembly operation with whichever hand they prefer.

## Materials and Methods

### Participants

Seventeen healthy subjects, from which one was left-handed, aged between 19 and 21 years volunteered as participants in the study. Due to abnormalities in the recording three subjects were excluded from further analysis, leaving a total of 14 participants. The study was restricted to male participants, both to exclude possible inter-gender differences and to replicate the selected job task more faithfully, since in the company that supported our research only males occupy the specific workplace under study. Participants did not report any past or present neurological or psychiatric conditions and were free of medication and psychoactive substances. They were instructed not to take any alcoholic drinks prior to, nor on the day of, participation in the study. All participants had normal or corrected-to-normal vision. They agreed to participate in the study and signed informed consent after reading the experiment summary in accordance with the *Declaration of Helsinki*. The Ethical Committee of the University of Kragujevac approved the study and procedures for the participants.

### Replication of the Workplace

**Figure 1:**
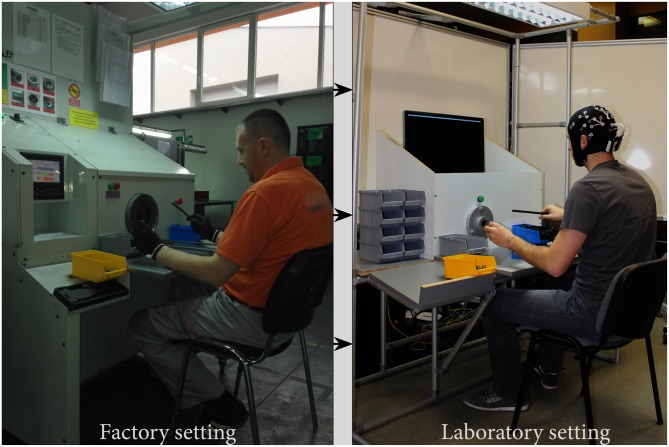
**Left image—Real workplace (replicated from our industrial partner); Right image—Replicated workplace**.

As we stated in the introduction, reliable EEG recording still relies on wet electrodes, limiting on-site industrial EEG recording. For that reason, we simulated the production process of the rubber hoses, which are used in hydraulic brake systems in the automotive industry, in a faithfully replicated workplace (Figure 1). Full-scale replica of the specific workplace was created at the laboratory of University of Kragujevac, in consultation with the car sub-component manufacturing company. In order to create a naturalistic environment, all major elements from the real factory settings have been included while preserving respective spatial ratios and replicating ambient conditions.

The laboratory was air-conditioned and microclimate conditions controlled, keeping the ambient temperature at 24 ± 1°C while the measured relative air humidity value was between 40% and 60%. The luminance at the real workplace was also replicated from the industrial settings, using the same lighting and maintaining the luminance value at 810 lx. Finally, the noise trace was obtained by recording sounds in the vicinity of the original production facility, using cardiodid condenser microphone AT2020USB (Audio-technica, Japan), and this was replayed during the experiments with an SW-HF 5.1 6000 surround multimedia speaker (Genius, Taiwan). The ambient (light, noise) and microclimate (temperature, humidity) condition values were obtained using multifunctional environmental meter device PCE-EM882 (PCE instruments, UK).

The experimental setup used in this study was similar to previously reported studies (Mijović et al., 2015a,b, 2016), while the experimental task and procedure were modified. For clarity, we will repeat the detailed experimental setup here.

### Simulated Assembly Operation

**Figure 2:**
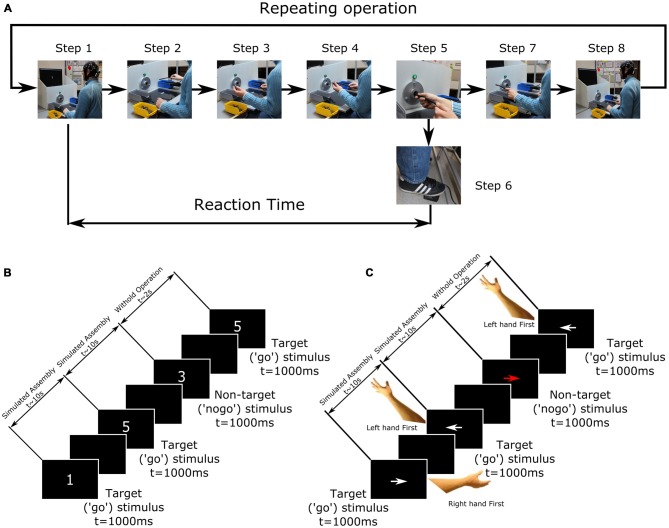
**(A)** Step by step representation of the simulated working process. Step 1—Stimulus presentation; Step 2—Taking the rubber hose; Step 3—Taking the metal part; Step 4—Placing metal part on the rubber hose; Step 5—Insertion of the uncompleted part inside the improvised machine opening; Step 6—Pressing the pedal in order to initiate the simulated crimping operation; Step 7—Placing the completed into the box with completed parts; Step 8—Waiting for the successive stimulus presentation. **(B)** Graphical representation of the Numbers Task. **(C)** Graphical Representation of the Arrows task.

In the production process, an operator carries out a crimping operation in order to join a metal extension to a rubber hose. This single operation, carried out in a sitting position, consists of eight simple steps (actions). Step-by-step simulated operation, carried out by participants in the replicated working environment, is graphically presented in Figure 2A and explained in detail further in the text.

The major production steps can be summarized as follows (Figure 2A): first, the information to initiate the simulated assembly operation is presented to the participant, in the form of visual stimulus (step 1, explained in detail in Section “Experimental Procedure”), upon which he is instructed to instantly initiate the operation by taking the metal part (step 2) and the rubber hose (step 3). Following this, participants should place the metal part on the hose (step 4) and place both inside the crimping machine (step 5). Once the rubber hose and metal part are correctly placed inside the opening, the industrial green lamp lights and presents a visual cue to the participant, informing him that the part has been correctly placed. Participant then proceed by promptly pressing the pedal, which initiates the improvised machine and replicates the real machines’ crimping sound with a duration of 3500 ms (step 6). The real crimping operation that would happen upon pressing the pedal was avoided, preserving its major aspects from operator’s perspective—the sound it produces and the cessation of which indicates the end of machine operation, analogously to the real case. Upon completion of the simulated crimping process, the participant removes the component and places it in the box with completed parts (step 7). Finally, following these steps, the participant sits still, waiting for the subsequent stimulus (step 8) indicating the next-in-line operation.

Although the assembly task consists of eight sub-actions, the whole operation lasts less than 10 s and a single operator completes between 2500–3000 elements during a work shift. Hence, this workplace represents a typical example of a repetitive, monotonous operational task in industrial assembly settings.

### Preparation

Each participant arrived to the laboratory at 9:00 a.m. Upon carefully reading the experiment summary and signing the informed consent for participation in the study, participants started the training session in order to gain familiarity with the task. Due to its simplicity, they were given 15 min for practicing, following which they confirmed their readiness to start the experiment. Finally, an EEG cap and amplifier were mounted on the participant’s head (as explained in the Section “EEG Recording”) and the recording started around 9:30 a.m.

### Experimental Procedure

During the experiment, at least two experimenters were constantly present in the laboratory in order to assure that experimental procedures were strictly followed. The experimenters were seated behind an opaque board (so that participants could not see them during the task) and they observed the participants through a red-blue-green (RGB) camera that recorded the entire experiment.

Participants were seated in a comfortable chair in front of an improvised workplace including the improvised machine (Figure 1). In order to extract the ERP component from continuous EEG recording, a single functional modification in the simulated assembly task was made. Simultaneously with the simulated assembly process, the participants were subjected to either the Numbers (Figure 2B) or Arrows (Figure 2C) task to prompt initiation of the assembly operation. Both tasks were presented on the 24” screen from a distance of approximately 100 cm in a balanced order across participants (with a 15 min break between the tasks). The screen was height adjustable and the center of the screen was set to be level with participants’ eyes. Upon presentation of the stimuli on the screen, the participants were instructed to complete the previously explained assembly operation (also graphically presented in Figure 2A).

All the stimuli were presented for 1000 ms on a black screen background. In both tasks the appearance of the stimuli was randomized, with the condition that forbade the two consecutive appearance of the “no-go” stimuli (digit “3” in Numbers, and red arrow in Arrows task). Additionally, in the Numbers tasks, five randomly allocated digit sizes were presented to increase the demands for processing the numerical value and to minimize the possibility that subjects would set a search template for some perceptual feature of the “no-go” trial (the digit “3”). Digit font sizes were 60, 80, 100, 120 and 140 in Arial text font (similar to Dockree et al., 2005). The main difference between the tasks is that in the Arrows tasks the participants were instructed to initiate the simulated operation with the right hand (step 2) if the white arrow was pointing to the right, or with the left hand (step 3) if pointing left (as depicted on Figure 2C), while in the Numbers task, the participants could freely choose between step 2 or step 3 (from the Figure 2A) upon seeing the digit. Each task consisted of 500 stimuli, where the probability of appearance of the “no-go” stimuli was set at 10% (50 in total), while the “go” stimuli were presented 450 times. The inter-stimulus interval (ISI) between two consecutive “go” stimuli was on average 11,240 ms (STD = 410 ms), while between “no-go” and following “go” stimuli the average ISI was 3210 ms (STD = 120 ms). The duration of the each task was around one and a half hours, upon which participants had a 15 min break, before starting the second task. Thus, the whole experiment lasted around 3 h and 15 min.

The task specifications were programmed in Simulation and Neuroscience Application Platform (SNAP)^1^, developed by the Swartz Center for Computational Neuroscience (SCCN). As explained in Bigdely-Shamlo et al. (2013) and Gramann et al. (2014), SNAP is a python-based experiment control framework that is able to send markers as strings to Lab Streaming Layer (LSL)^2^. LSL is a real-time data collection and distribution system that allows multiple continuous data streams as well as discrete marker timestamps to be acquired simultaneously in an eXtensible Data Format (XDF)^3^. This data collection method provides synchronous, precise recording of multi-channel, multi-stream data that is heterogeneous in both type and sampling rate (Bigdely-Shamlo et al., 2013; Gramann et al., 2014), and is obtained via a local area network (LAN).

### EEG Recording

EEG data acquisition was performed using the SMARTING (mBrainTrain, Serbia) wireless EEG system, with a sampling frequency of 500 Hz and 24-bit data resolution. The small and lightweight EEG amplifier (85 × 51 × 12 mm, 60gr) is tightly connected to a 24-channel electrode cap (Easycap, Germany) at the occipital site of the participant’s head, using an elastic band. The connection between the EEG amplifier and recording computer was obtained using a Bluetooth connection, and the data were streamed to the described LSL recorder. The design of the cap-amplifier unit ensured minimal isolated movement of individual electrodes, cables, or the amplifier, which strongly reduced electromagnetic interference and movement artifacts. Further, the small dimensions of the recording system provided full mobility and comfort to the participants, as movement constraints were not imposed. The electrode cap contained sintered Ag/AgCl electrodes that were placed based on the international 10–20 System: Fp1, Fp2, Fz, F7, F8, FC1, FC2, Cz, C3, C4, T7, T8, CPz, CP1, CP2, CP5, CP6, TP9, TP10, Pz, P3, P4, O1 and O2. The electrodes were referenced to FCz and the ground electrode was AFz. Before initiation of the experiments, the experimental procedure imposed that the electrode impedances must be below the 5 kΩ value, which was confirmed by the device acquisition software.

### ERP Processing

EEG signal processing was performed offline using EEGLAB (Delorme and Makeig, 2004) and MATLAB (Mathworks Inc., Natick, MA, USA). EEG data were first bandpass filtered in the 1–35 Hz range, following which the signals were re-referenced to the average of the mastoid channels (Tp9 and Tp10). Further, an extended infomax Independent Component Analysis (ICA) was used to semi-automatically attenuate contributions from eye blink and (sometimes) muscle artifacts (as explained in Viola et al., 2009; De Vos et al., 2010, 2011). After this data preprocessing, ERP epochs were extracted from −200 to 800 ms with respect to timestamp values of “go” and “no-go” stimuli indicated by the SNAP software. Baseline values were corrected by subtracting mean values for the period from −200 to 0 ms from the stimuli. The identified electrode sites of interest for the ERP analysis in this study were Fz, Cz, CPz and Pz, as the P300 component is most prominent over the central and parieto-central scalp locations (Picton, 1992).

For the “no-go” condition we extracted and averaged the ERPs across the trials. For the “go” condition, the ERPs that preceded the “no-go” condition were calculated. Following these steps, the grand average (GA) ERPs across participants were formed. Further, the P300 amplitude was calculated for both “go” and “no-go” conditions and for each experimental condition, using mean amplitude measure (Luck, 2005) in the time window from 350 to 450 ms, with regard to the time stamps of the stimuli. Finally, the statistical analysis on the obtained results was carried out.

### Reaction Times

As already stated in Section “Experimental Procedure”, our experimental design did not allow subjects to react with the button press upon seeing the visual “go” stimulus. Therefore, the RT could not be measured in the traditional fashion, as the time elapsed between the stimulus presentation and the response by the participants (usually executed with the right index finger). Instead, the RTs here were measured as the time elapsed between the stimulus presentation (step 1) and the pedal press (step 6 from the Section “Preparation”, also depicted on the Figure 2A). The pedal used in our study was actually a modified mouse button and it was connected to the recording computer via USB connection. As LSL is capable of real-time recording of the timestamps of the mouse button press, it enabled us to gather precise information regarding the time when pedal was pressed. This allows the calculation of RTs, as the difference between timestamps from stimulus presentation (operation initiation) and the beginning of the machine simulated crimping process.

### Error Processing

Errors of omission were classified as the errors occurring when participants did not respond to the appearance of the “go” stimuli. The commission errors processing was challenging, since our task did not require a speeded button press and therefore, the errors of commission were difficult to interpret. In fact, the most obvious classification of commission errors would be when participants completely execute the simulated operation upon appearance of the “no-go” stimuli. However, it is important to note that participants sometimes made slight movements upon appearance of the “no-go” stimuli (in sense that they showed intention to initiate the action) and then they inhibited the response upon realization that it was a “no-go” stimulus. This kind of error we classified as near-misses. The identification of the near misses and commission errors was conducted initially by the experimenters in the room and subsequently confirmed in an off-line analysis, by replaying the videos recorded with the RGB camera during the experiment.

#### Statistical Analysis

The statistical analysis was performed using IBM SPSS software. The ERPs used for statistical analysis included all ERPs related to the “no-go” condition and 50 ERPs related to “go” preceding the “no-go” condition. The 4 × 2 × 2 × 2 repeated measures analysis of variance (ANOVA) was conducted with Site (Fz, Cz, CPz and Pz), Task (Arrows vs. Numbers) and Condition (“go/no-go”) as within subject factors and Order of presentation (first vs. second) as between-subject factor. Additionally, a 2 × 2 ANOVA comparing RTs across Task (Arrows vs. Numbers) as within subject factors and Order of presentation (first vs. second) as between subject factor was conducted. Finally, we carried out a 2 × 2 ANOVA comparing near misses across Task (Arrows vs. Numbers) as within subject factors and Order of presentation (first vs. second) as between subject factor. Greenhouse-Geissser corrections (FG) were applied where necessary. Since the participants did not make any omission errors and only seven commission errors occurred across the participants they were exempted from further statistical analysis.

## Results

### Behavioral Results

#### Reaction Times

The 2 × 2 ANOVA comparing RTs across Task (Arrow vs. SART) condition as within subject factor and Order of presentation (first vs. second) as between subject factor revealed neither significant main effects, nor interaction effects.

#### Errors

As stated in the “Materials and Methods” Section (Section “Statistical Analysis”), the participants did not make any omission errors and the low number of omission errors were not statistically analyzed. However, regarding near-misses, the ANOVA revealed only a significant effect of task (*F*_(1,8)_ = 11.9, *p* < 0.01, *η* = 0.60) with more near-misses occurring in the Numbers compared to the Arrows task.

### ERP Results

**Figure 3:**
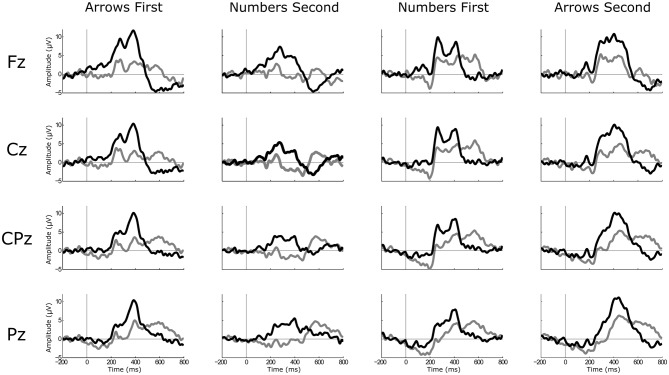
**Graphical representation of the grand average (GA) event-related potentials (ERPs) for each task and each electrode location under study.** The black line represents the “go” condition, while the gray line depicts the GA ERPs for the “no-go” condition.

The GA ERPs for each task (Arrows and Numbers), each condition (“go/no-go”) and each electrode site under study (Fz, Cz, CPz and Pz) are depicted in Figure 3.

The 4 × 2 × 2 × 2 ANOVA revealed that the ERPs differed depending on the condition (Go/No-Go: *F*_(1,12)_ = 5.99, *p* < 0.05, η = 0.33), the task (Task: *F*_(1,12)_ = 17.06, *p* < 0.001, *η* = 0.59), the order of presentation (Order of presentation: *F*_(1,12)_ = 15.635, *p* < 0.01, *η* = 0.57) and across the scalp (Site: *F*_(1.48,17.75)_ = 5.352, *p* < 0.05, *η* = 0.31). Namely, the P300 amplitudes elicited for “go” trials were higher than for “no-go” trials (*M* = 5.73, *SD* = 1.47; *M* = 2.25, *SD* = 1.41, respectively). Further, the Arrow task produced higher amplitudes in comparison to Numbers (*M* = 5.24, *SD* = 1.11; *M* = 2.73, *SD* = 1.46, respectively). The P300 amplitudes elicited with regard to the Order of presentation demonstrated higher amplitudes for whichever task was presented first in comparison to second task (*M* = 5.11, *SD* = 1.31; *M* = 2.86, *SD* = 1.54, respectively). Finally, amplitudes elicited at Pz were significantly higher than the amplitudes at the other three sites and amplitudes at CPz site were higher than at Cz and Fz sites at the *p* < 0.05 level. All the other comparisons were significant in the same direction apart from the Fz-Cz difference.

**Figure 4:**
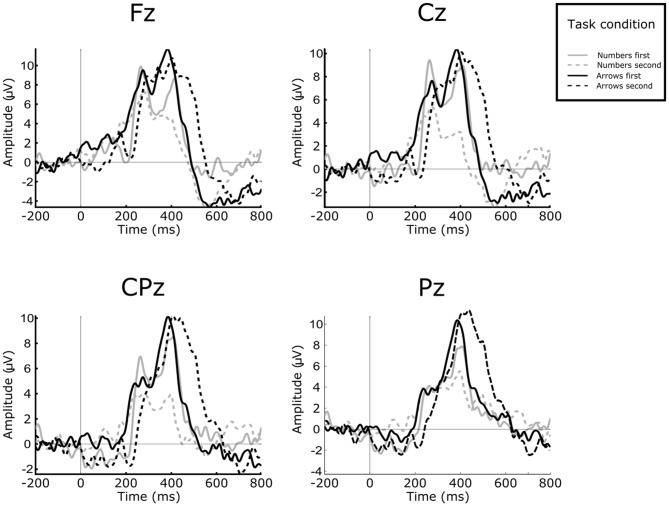
**The GA ERPs elicited for “go” condition in all four experimental conditions.** ERPs elicited for The Numbers task are represented with the gray color, while the ERPs elicited in the Arrows task are depicted with the black color. The full line represents that the task was presented as a first task and the dashed line if the task was presented as second task.

Figure 4 depicts the GA ERPs elicited over all four electrode sites under study for the “go” condition.

**Figure 5:**
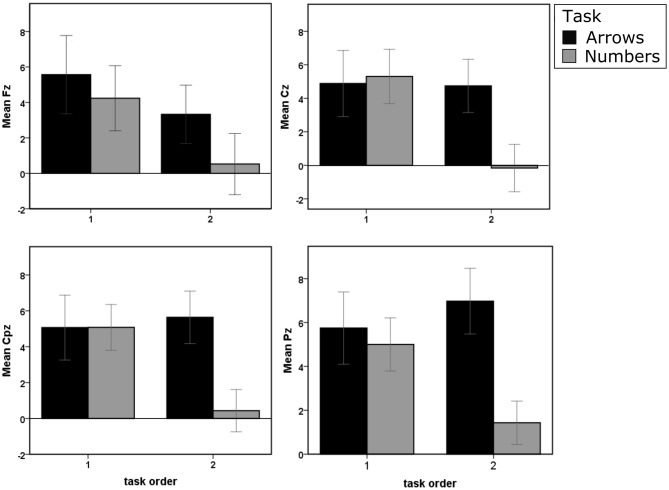
**The amplitude values for all four electrode sites and for all experimental conditions.** The black color depicts the Arrows task, while the Numbers task is represented with the gray color. The error bars represents ±2 SE.

The P300 amplitude differences for all four sites and depending on the task representation order are presented in Figure 5.

## Discussion

The present study investigated whether operators’ attention is enhanced when they are instructed with which hand to initiate the manual assembly operation, as compared to spontaneous and free choice of preferred hand. The attention was assessed through the P300 amplitude, as it is widely accepted that the P300 amplitude is positively related to the human level of attention (Ford et al., 1994; Polich, 2007; De Vos et al., 2014). For this aim we simulated a manual assembly operation, where we provided the participants with two distinct psychological tasks (Numbers and Arrows) simultaneously with the simulated operation.

The P300 components’ amplitude was significantly higher in magnitude for the frequent “go” (target), than for the infrequent “no-go” condition (as presented on the Figure 3). This finding is in contrast to the majority of previously reported studies where an infrequent target condition elicits a higher magnitude of the P300 amplitude, since the participants are usually required to note the occurrence of infrequent targets by button press or by silent counting (Strüber and Polich, 2002). On the other hand, in our task target stimuli were the frequent ones, as the continuity of operation in manual assembly is essential, while the participants were instructed just to sit still and with no actions during the infrequent “no-go” condition. As such, it is not surprising that the lower magnitude of the P300 amplitude were elicited in infrequent non-target condition, as passive stimulus processing induces smaller P300 amplitudes than active tasks (Polich, 2007). This was also supported by the results from the study of Potts et al. (2004), where they reported that the P300 amplitude was larger in frequent “go” condition as compared to rare non-target condition in the task where the ratio between “go” and “no-go” condition was 80/20. Moreover, it was found that the ISI between target stimuli influences the P300 amplitude, in the sense that a short ISI leads to decreased amplitude, while relatively long ISIs elicit the higher P300 amplitude, which is the case even in the single-stimulus paradigm (Strüber and Polich, 2002; Polich, 2007). This was the case also in our study, since the ISI was relatively long (approximately 11 s) and we believe that it was suitable for eliciting the P300 amplitude even in the frequent target condition.

The main finding of the present study is that the P300 amplitude was considerably higher in magnitude when participants were instructed with which hand to initiate the simulated assembly operation, as compared to the case when participants could freely choose the preferred hand for the operation initiation. This may not be surprising, since in the choice reaction task (Arrows) participants were subjected to slightly higher demands of the incoming stimuli evaluation, as they were un-aware of the direction in which the white arrow stimuli would point. On the other hand, the digit stimulus carries considerably lower information, as participants are required just to make distinction whether it is a “go” or “no-go” stimulus and to perform their action accordingly, i.e., the participants may stop evaluating the content of the stimuli after some time. Therefore, the response selection requirements during the Arrows task are substantially higher than in Numbers task, which may lead to increased P300 amplitude in the condition which required instructed responding from the participants (Verleger et al., 2005, 2014). Following this finding, it may be proposed that the workers on repetitive and monotonous assembly tasks should not receive information solely on whether they should initiate the operation or not, but it should be beneficial if they receive information that carries slightly higher cognitive demands. In fact, the task that consisted of the stimuli with the higher cognitive demands induced the higher P300 amplitude, which may be related to the attention of the worker for the task in hand. An important notion, however, is that there is possibility that the P300 amplitude in this study does not reflect solely the attention level of a worker, but it also may be influenced by the different cognitive demands of the tasks. For that reason, it is important to further investigate whether the P300 amplitude was influenced by the presented task demands, or it was solely related to the attention of the workers.

Interestingly, although it was expected that the RTs could differ between the two tasks, this was not the case in our study. One of the possible reasons for the absence of the response time effect could be the methodology used for the RTs calculation. In fact, the time period for RT calculation is much longer than in the conventional studies, where a speeded response from the participants is expected. Apart from that, the RT calculation includes several coordinated hand movements before the foot switch is pressed. All of these could induce a large variation within and between subject conditions, which may induce inaccuracy of the RT methodology used in this study. Further, with regard to behavioral measurements, the number of commission errors was relatively low and did not differ between the tasks. However, there was significantly higher amount of near-misses in the Numbers than in the Arrows task. The fact that there was larger number of near-misses in the Numbers task may be expected, as the Arrows task imposes a higher workload on the participants, due to the higher response selection requirements, and as it was previously reported, the errors and mental workload are related according to a U-shaped curve (Desmond and Hoyes, 1996).

Although we showed that the Arrows task produced a higher P300 amplitude than the Numbers task, one could argue about the selection of the tasks, as the stimulus type between task conditions significantly differed (digits vs. arrows). The main reason for not investigating the difference between instructed and non-instructed condition with the same type of stimuli was the avoidance of the interference effect (Pashler, 1994). In fact, if only stimuli from Numbers task were used and dedicated the directions to specific digits in the hand instructing task (e.g., odd numbers means left and even numbers right hand first), it would be highly likely that the memory would strongly influence the attention processing. On the other hand, if we only used the Arrows stimuli type, an undesired bias would be included in the condition when participants could initiate the operation with their preferred hand. An additional concern is whether the two distinct psychological tests trigger different attentional resources, given that they are composed of different stimulus types and that the Arrows task alternates the response hand, while in the Numbers task participants could respond with whichever hand they preferred. The answer to this doubt could be found in premotor theory of attention (Rizzolatti et al., 1994), which states that attention orienting processes are triggered during uni-manual response preparation and that the orienting processes are assumed to be equivalent to the processes elicited during instructed endogenous shifts of spatial attention (Eimer et al., 2005). Moreover, Ranzini et al. (2009) also used the tasks with Arabic digits and Arrows and they demonstrated that processes evoked by these cues are alike and that the volitional and non-volitional attentional shifts rely on the same fronto-parietal brain networks. Thus, both Numbers and Arrows tasks should evoke the same cognitive resources of attention, which gives legitimacy to the choice of the tasks used in this study.

One of the limitations of the present study is that it was conducted in a simulated working environment, instead of a real factory setting. The main reason for this was usage of the wet-electrode EEG recording system, which is still uncomfortable for application in actual industrial environments. Nevertheless, we replicated both the spatial dimensions and ambient conditions and performed the wearable EEG study, demonstrating its applicability for the investigation of covert cognitive processes in naturalistic environments for HF/E studies. Another limitation is that, simultaneously with the simulated operation, we used two distinct psychological tests, with the aim of eliciting the P300 ERP component. Although it could be argued that psychological tests could interfere with the simulated operation, an important notion is that the assembly workers should be provided with timely information regarding the performed operation (Stork and Schubö, 2010). Therefore, we believe that this modification did not significantly differ from the actual assembly operation in industrial environments. Moreover, in naturalistic settings it is usually hard to isolate and analyze the specific cognitive process, since they should first be evoked and co-occurring cognitive factors should be isolated (Bulling and Zander, 2014). Thus, this modification in the information presentation to the participants was necessary in order to elicit the anticipated P300 ERP component during the simulated assembly operation. Unfortunately, the present study is unable to compare brain responses between self-paced (as in this specific workplace) and externally paced work routines that we used in our study. This issue should be addressed in future studies.

The present study demonstrated that wearable EEG recording could be beneficial for task design in HF/E studies. Future studies should investigate whether the reported findings also hold for similar job positions, which are monotonous and repetitive in nature but require continuous focus of the worker on the industrial task (e.g., quality control tasks). Although the present study utilized wearable EEG in a faithfully replicated workplace environment, it seems that it is just a matter of time until EEG systems will be willingly accepted for everyday use (van Erp et al., 2012; Mihajlovic et al., 2015). This could even lead to the application of passive brain-computer interfaces, which could be used for real-time assessment of the cognitive user states in industrial environments (Zander and Kothe, 2011). Nevertheless, the fact that it is nowadays possible to investigate brain dynamics during natural movements (without imposing movements constraints) of the recorded individual brings us a step closer to the guiding principle of the neuroergonomics, that is, to investigate how the brain carries out the complex tasks of everyday life and not just simplified and artificial tasks in the laboratory settings (Parasuraman and Rizzo, 2006).

## Conclusion

Comparing monotonous (“go/no-go”) Numbers task to the choice-reaction (Arrows) task, which instructs the participants with which hand to commence the assembly operation, the present study indicates that the latter is more suitable to preserve participants’ attention during the initiation of externally-paced assembly task. This finding was achieved through investigation of the ERP waveform, where it was found that the P300 amplitude, which is related to the level of attention, was enhanced in the task that instructed the participants with which hand to initiate the simulated assembly operation. This study demonstrated the potential benefits of introducing the EEG measurements in the industrial task design, as from the presented results it may be concluded that in in monotonous assembly tasks, instructed responding, or a similar method of engagement, should be imposed on operators, since it is indicated that additional engagement enhances the worker’s attention.

## Author Contributions

Study design and protocols were created by PM, VK, MDeV, BJ, IM and IG. Data acquisition was performed by PM and IG. Data analysis was performed by PM, VK, MDeV, IM and IG. The data interpretation was performed by PM, VK, BJ, IM and IG. The manuscript was written by PM and VK and critical editing was performed by MDeV, BJ, IM and IG. Final approval of the version was obtained from all co-authors. All co-authors agree on all aspects of the work and ensure that questions related to the accuracy and integrity of any part of the submitted work are appropriately investigated and resolved.

## Conflict of Interest Statement

IG is associated with the mBrainTrain Company, supplier of wireless EEG system “SMARTING” used in this study. However, no financial or other conflicting interests arise from this fact. The other authors declare that the research was conducted in the absence of any commercial or financial relationships that could be construed as a potential conflict of interest.

## Footnotes

^1^https://github.com/sccn/SNAP

^2^https://code.google.com/p/labstreaminglayer/

^3^https://code.google.com/p/xdf/

## References

[B1] Ayaz H. Onaral B. Izzetoglu K. Shewokis P. A. McKendrick R. Parasuraman R. (2013). Continuous monitoring of brain dynamics with functional near infrared spectroscopy as a tool for neuroergonomic research: empirical examples and a technological development. Front. Hum. Neurosci. 7:871. 10.3389/fnhum.2013.00871 24385959 PMC3866520

[B2] Ayaz H. Shewokis P. A. Bunce S. Izzetoglu K. Willems B. Onaral B. (2012). Optical brain monitoring for operator training and mental workload assessment. Neuroimage 59, 36–47. 10.1016/j.neuroimage.2011.06.023 21722738

[B3] Ayaz H. Willems B. Bunce B. Shewokis P. A. Izzetoglu K. Hah S. (2010). “Cognitive workload assessment of air traffic controllers using optical brain imaging sensors,” in Advances in Understanding Human Performance: Neuroergonomics, Human Factors Design and Special Populations, eds Marek T. Karwowski W. Rice V. (Boca Raton, FL: CRC press), 21–31.

[B4] Bigdely-Shamlo N. Kreutz-Delgado K. Robbins K. Miyakoshi M. Westerfield M. Bel-Bahar T. (2013). “Hierarchical event descriptor (HED) tags for analysis of event-related EEG studies,” in Global Conference on Signal and Information Processing (GlobalSIP), Austin, TX, 1–4.

[B5] Bulling A. Zander T. O. (2014). Cognition-aware computing. IEEE Perv. Comp. 13, 80–83. 10.1109/mprv.2014.42

[B6] De Vos M. De Lathauwer L. Van Huffel S. (2011). Spatially constrained ICA algorithm with an application in EEG processing. Sign. Process. 91, 1963–1972. 10.1016/j.sigpro.2011.02.019

[B7] De Vos M. Gandras K. Debener S. (2014). Towards a truly mobile auditory brain-computer interface: exploring the P300 to take away. Int. J. Psychophysiol. 91, 46–53. 10.1016/j.ijpsycho.2013.08.010 23994208

[B8] De Vos M. Riès S. Vanderperren K. Vanrumste B. Alario F. X. van Huffel S.. (2010). Removal of muscle artifacts from EEG recordings of spoken language production. Neuroinformatics 8, 135–150. 10.1007/s12021-010-9071-0 20480401

[B9] Debener S. Minow F. Emkes R. Gandras K. de Vos M. (2012). How about taking a low-cost, small and wireless EEG for a walk? Psychophysiology 49, 1617–1621. 10.1111/j.1469-8986.2012.01471.x 23013047

[B10] Delorme A. Makeig S. (2004). EEGLAB: an open source toolbox for analysis of single-trial EEG dynamics including independent component analysis. J. Neurosci. Methods 134, 9–21. 10.1016/j.jneumeth.2003.10.009 15102499

[B11] Desmond P. A. Hoyes T. W. (1996). Workload variation, intrinsic risk and utility in a simulated air traffic control task: evidence for compensatory effects. Saf. Sci. 22, 87–101. 10.1016/0925-7535(96)00008-2

[B12] Dockree P. M. Kelly S. P. Robertson I. H. Reilly R. B. Foxe J. J. (2005). Neurophysiological markers of alert responding during goal-directed behavior: a high-density electrical mapping study. Neuroimage 27, 587–601. 10.1016/j.neuroimage.2005.05.044 16024257

[B13] Donchin E. (1981). Surprise!… surprise? Psychophysiology 18, 493–513. 10.1111/j.1469-8986.1981.tb01815.x 7280146

[B14] Donkers F. C. van Boxtel G. J. (2004). The N2 in go/no-go tasks reflects conflict monitoring not response inhibition. Brain Cogn. 56, 165–176. 10.1016/j.bandc.2004.04.005 15518933

[B15] Eimer M. Forster B. Van Velzen J. Prabhu G. (2005). Covert manual response preparation triggers attentional shifts: ERP evidence for the premotor theory of attention. Neuropsychologia 43, 957–966. 10.1016/j.neuropsychologia.2004.08.011 15716166 PMC2254498

[B16] Ford J. M. Sullivan E. V. Marsh L. White P. M. Lim K. O. Pfefferbaum A. (1994). The relationship between P300 amplitude and regional gray matter volumes depends upon the attentional system engaged. Electroencephalogr. Clin. Neurophysiol. 90, 214–228. 10.1016/0013-4694(94)90093-0 7511503

[B18] Gramann K. Ferris D. P. Gwin J. Makeig S. (2014). Imaging natural cognition in action. Int. J. Psychophysiol. 91, 22–29. 10.1016/j.ijpsycho.2013.09.003 24076470 PMC3983402

[B19] Gramann K. Gwin J. T. Ferris D. P. Oie K. Jung T. P. Lin C. T.. (2011). Cognition in action: imaging brain/body dynamics in mobile humans. Rev. Neurosci. 22, 593–608. 10.1515/RNS.2011.047 22070621

[B21] Johnson R. (1988). “The amplitude of the P300 component of the event-related potential: review and synthesis,” in Advances in Psychophysiology: A Research Annual, (Vol. 3), eds Ackles P. Jennings J. R. Coles M. G. H. (Greenwich, CT.: JAI press), 69–137.

[B22] Kletz T. A. (2001). An Engineer’s View of Human Error. (Vol. 3), Rugby: Institution of Chemical Engineers.

[B23] Luck S. J. (2005). An Introduction to the Event-Related Potential Technique. Cambridge, MA: MIT Press.

[B24] Mehta R. K. Parasuraman R. (2013). Neuroergonomics: a review of applications to physical and cognitive work. Front. Hum. Neurosci. 7:889. 10.3389/fnhum.2013.00889 24391575 PMC3870317

[B25] Michalos G. Makris S. Papakostas N. Mourtzis D. Chryssolouris G. (2010). Automotive assembly technologies review: challenges and outlook for a flexible and adaptive approach. CIRP J. Manuf. Sci. Tech. 2, 81–91. 10.1016/j.cirpj.2009.12.001

[B26] Mihajlovic V. Grundlehner B. Vullers R. Penders J. (2015). Wearable, wireless EEG Solutions in daily life applications: what are we missing? IEEE J. Biomed. Health Inform. 19, 6–21. 10.1109/jbhi.2014.2328317 25486653

[B27] Mijović P. Ković V. De Vos M. Mačužić I. Todorović P. Jeremić B.. (2016). Towards continuous and real-time attention monitoring at work: reaction times versus brain response. Ergonomics [Epub ahead of print]. 10.1080/00140139.2016.1142121 26772445

[B28] Mijović P. Ković V. Mačužić I. Jeremić B. Todorović P. Milovanović M. (2015a). “Do micro-breaks increase the attention level of an assembly worker? An ERP study,” in Proceedings of 6th International Conference of Applied Human Factors and Ergonomics (AHFE 2015), Las Vegas, NV.

[B29] Mijović P. Milovanović M. Minović M. Mačužić I. Ković V. Gligorijević I. (2015b). “Towards creation of implicit HCI model for prediction and prevention of operators’ error,” in Human-Computer Interaction: Interaction Technologies, ed. Kurosu M. (Cham: Springer International Publishing), 341–352.

[B30] Parasuraman R. (1990). “Event-related brain potentials and human factors research,” in Event-Related Brain Potentials: Basic Issues and Applications, eds Rohbaugh J. Parasuraman R. Johnson R. (New York, NY: Oxford University Press), 279–300.

[B31] Parasuraman R. (2003). Neuroergonomics: research and practice. Theor. Issues Ergon. Sci. 4, 5–20. 10.1080/14639220210199753

[B32] Parasuraman R. (2011). Neuroergonomics brain, cognition and performance at work. Curr. Dir. Psychol. Sci. 20, 181–186. 10.1177/0963721411409176

[B33] Parasuraman R. Rizzo M. (Eds.) (2006). Neuroergonomics: The Brain at Work. New York, NY: Oxford University Press.

[B35] Parasuraman R. Wilson G. F. (2008). Putting the brain to work: neuroergonomics past, present and future. Hum. Factors 50, 468–474. 10.1518/001872008x288349 18689055

[B36] Pashler H. (1994). Dual-task interference in simple tasks: data and theory. Psych. Bull. 116, 220–244. 10.1037/0033-2909.116.2.220 7972591

[B37] Picton T. W. (1992). The P300 wave of the human event-related potential. J. Clin. Neurophysiol. 9, 456–479. 10.1097/00004691-199210000-00002 1464675

[B38] Polich J. (2007). Updating P300: an integrative theory of P3a and P3b. Clin. Neurophysiol. 118, 2128–2148. 10.1016/j.clinph.2007.04.019 17573239 PMC2715154

[B39] Polich J. Kok A. (1995). Cognitive and biological determinants of P300: an integrative review. Biol. Psychol. 41, 103–146. 10.1016/0301-0511(95)05130-9 8534788

[B40] Potts G. F. Patel S. H. Azzam P. N. (2004). Impact of instructed relevance on the visual ERP. Int. J. Psychophysiol. 52, 197–209. 10.1016/j.ijpsycho.2003.10.005 15050377

[B41] Ranzini M. Dehaene S. Piazza M. Hubbard E. M. (2009). Neural mechanisms of attentional shifts due to irrelevant spatial and numerical cues. Neuropsychologia 47, 2615–2624. 10.1016/j.neuropsychologia.2009.05.011 19465038

[B42] Rizzolatti G. Riggio L. Sheliga B. M. (1994). Space and selective attention. Atten. Perform. XV 15, 231–265.

[B43] Robertson I. H. Manly T. Andrade J. Baddeley B. T. Yiend J. (1997). Oops!’: performance correlates of everyday attentional failures in traumatic brain injured and normal subjects. Neuropsychologia 35, 747–758. 10.1016/s0028-3932(97)00015-8 9204482

[B44] Scerbo M. (2006). “Adaptive automation,” in Neuroergonomics: The Brain at Work, eds Parasuraman R. Rizzo M. (New York, NY: Oxford University Press), 239–252.

[B45] Sheridan T. B. Parasuraman R. (2005). Human-automation interaction. Rev. Hum. Fact. Ergon. 1, 89–129. 10.1518/155723405783703082

[B46] Spath D. Braun M. Meinken K. (2012). “Human factors in manufacturing,” in Handbook of Human Factors and Ergonomics, ed. Salvendy G. (New Jersey, NJ: John Wiley & Sons), 1643–1666.

[B47] Stork S. Schubö A. (2010). Human cognition in manual assembly: theories and applications. Adv. Eng. Inform. 24, 320–328. 10.1016/j.aei.2010.05.010

[B48] Strüber D. Polich J. (2002). P300 and slow wave from oddball and single-stimulus visual tasks: inter-stimulus interval effects. Int. J. Psychophysiol. 45, 187–196. 10.1016/s0167-8760(02)00071-5 12208526

[B49] Tang A. Owen C. Biocca F. Mou W. (2003). “Comparative effectiveness of augmented reality in object assembly,” in Proceedings of the SIGCHI Conference on Human Factors in Computing Systems. (New York, NY: ACM), 73–80.

[B50] Tompkins J. A. White J. A. Bozer Y. A. Tanchoco J. M. A. (2010). Facilities Planning. New Jersey, NJ: John Wiley & Sons.

[B51] van Erp J. B. Lotte F. Tangermann M. (2012). Brain-computer interfaces: beyond medical applications. IEEE Comput. 45, 26–34. 10.1109/mc.2012.107

[B52] Verleger R. Baur N. Metzner M. F. Šmigasiewicz K. (2014). The hard oddball: effects of difficult response selection on stimulus-related P3 and on response-related negative potentials. Psychophysiology 51, 1089–1100. 10.1111/psyp.12262 24981371

[B53] Verleger R. Jaśkowski P. Wascher E. (2005). Evidence for an integrative role of P3b in linking reaction to perception. J. Psychophysiol. 19, 165–181. 10.1027/0269-8803.19.3.165

[B54] Viola F. C. Thorne J. Edmonds B. Schneider T. Eichele T. Debener S. (2009). Semi-automatic identification of independent components representing EEG artifact. Clin. Neurophysiol. 120, 868–877. 10.1016/j.clinph.2009.01.015 19345611

[B55] Warm J. S. Matthews G. Parasuraman R. (2009). Cerebral hemodynamics and vigilance performance. Mil. Psych. 21, S75–S100. 10.1080/08995600802554706

[B57] Warm J. S. Parasuraman R. (2006). “Cerebral hemodynamics and vigilance,” in Neuroergonomics: The Brain at Work, eds Parasuraman R. Rizzo M. (New York, NY: Oxford University Press), 146–158.

[B56] Warm J. S. Parasuraman R. Matthews G. (2008). Vigilance requires hard mental work and is stressful. Hum. Factors 50, 433–441. 10.1518/001872008x312152 18689050

[B58] Wascher E. Heppner H. Hoffmann S. (2014). Towards the measurement of event-related EEG activity in real-life working environments. Int. J. Psychophysiol. 91, 3–9. 10.1016/j.ijpsycho.2013.10.006 24144635

[B59] Zander T. O. Kothe C. (2011). Towards passive brain-computer interfaces: applying brain-computer interface technology to human-machine systems in general. J. Neural Eng. 8:025005. 10.1088/1741-2560/8/2/025005 21436512

